# Effectiveness and Tolerability of Once-Weekly GLP-1 Receptor Agonists in Clinical Practice: A Focus on Switching Between Once-Weekly Molecules in Type 2 Diabetes

**DOI:** 10.3389/fendo.2022.892702

**Published:** 2022-07-15

**Authors:** Giulia Di Dalmazi, Sara Coluzzi, Maria Pompea Antonia Baldassarre, Amr Ghit, Giusi Graziano, Maria Chiara Rossi, Beatrice Ciappini, Marica Milo, Federica Carrieri, Antonio Nicolucci, Agostino Consoli, Gloria Formoso

**Affiliations:** ^1^ Department of Medicine and Aging Sciences, “G. d’Annunzio” University of Chieti-Pescara, Chieti, Italy; ^2^ Center for Advanced Studies and Technology (CAST), “G. d’Annunzio” University of Chieti-Pescara, Chieti, Italy; ^3^ Endocrinology and Metabolic Disease Clinic of Pescara, Pescara, Italy; ^4^ CORESEARCH-Center for Outcomes Research and Clinical Epidemiology, Pescara, Italy

**Keywords:** effectiveness, GLP-1 receptor agonists, dulaglutide, once-weekly exenatide, once-weekly semaglutide, real-world evidence, type 2 diabetes mellitus

## Abstract

**Aims:**

This study aims to evaluate the effectiveness and tolerability of once-weekly glucagon-like peptide receptor agonists (OW GLP-1RAs) and to assess the clinical benefits of switching from one GLP-1RA to another (switchers) in a routine clinical setting.

**Materials and Methods:**

This is a retrospective, real-world cohort study, based on electronic medical records utilized in one Italian diabetes clinic. Estimated mean changes in HbA1c and body weight after 6 and 12 months from the first prescription of a long-acting GLP1-RA were evaluated using longitudinal linear mixed models for repeated measures. The effectiveness of the three long-acting GLP1-RAs was compared separately in the GLP1-RA naive and switchers cohorts, after propensity score adjustment.

**Results:**

Initiating a long-acting GLP1-RA was associated with statistically significant improvements in HbA1c (−1%) and body weight (−2.6 kg) after 6 months, and benefits were maintained after 12 months. In GLP1-RA naive cohort, semaglutide showed the largest effect on HbA1c (−1.55%; 95%CI, −1.77;−1.34) and body weight (−3.76 kg; 95%CI, −5.05;−2.47) at 6 months, maintained at 12 months (−1.55%; 95%CI, −1.82;−1.28 and −6.29 kg; 95%CI, −7.94;−4.63). In the switchers’ cohort, statistically significant reductions at 6 months in HbA1c and body weight were documented with semaglutide and dulaglutide only, with semaglutide associated with the most marked reduction (−0.84%; 95%CI, −1.03;−0.65 and −3.43 kg; 95%, −4.67;−2.19). Dropout rates were 9.2%, 28.5%, and 41.7% in semaglutide, dulaglutide, and exenatide groups, respectively.

**Conclusions:**

The effectiveness and tolerability of the OW GLP-1RAs in the real world were documented. Semaglutide was associated with the highest response without impact on safety. Clinical improvements were obtained even in switchers, especially in those switching to semaglutide.

## Introduction

In the treatment of type 2 diabetes mellitus (T2DM), glucagon-like peptide 1 receptor agonists (GLP-1RAs) therapy has demonstrated effectiveness in reducing glycated hemoglobin (HbA1c), inducing body weight loss, reducing the risk of hypoglycemia, and protecting patients from cardiovascular diseases (1). Moreover, a meta-analysis of cardiovascular outcome trials (CVOTs) in patients with T2DM showed that GLP-1RAs improve cardiovascular and renal outcomes and reduce mortality rates (2).

In diabetic patients, once-weekly (OW) GLP-1RAs (dulaglutide, OW exenatide, and OW semaglutide) have a greater impact on decreasing HbA1c and body weight and are associated with fewer side effects as compared to molecules with shorter half-life (lixisenatide and liraglutide) (1).

Of note, solid evidence from randomized clinical trials (RCTs) confirms the efficacy and safety of OW GLP-1RAs as a complementary treatment to metformin (3, 4). Besides, GLP-1RAs were recommended by the American Diabetes Association (ADA) and the European Association for the Study of Diabetes (EASD) as first-line injectable therapy and second-line therapy when metformin fails to manage glucose levels (5). However, GLP-1RAs use is still relatively restricted worldwide probably due to physicians’ distrust on data gathered only from RCTs (6).

In a clinical setting, in order to maintain patients on effective therapies with proven cardiovascular benefits, the switch between GLP-1RAs may be considered when the molecule in use is unable to provide the desired glycemic control and/or weight loss, or when adverse effects occur (7, 8).

To date, just a few phase III RCTs directly compared the efficacy of different GLP-1RAs (9–11). In addition, direct comparisons among GLP-1RAs have been relatively limited in real world data (RWD) (12–15). Thus, there is the need for a real-world setting comparative analysis of available OW GLP-1RAs in order to support data from RCTs and to provide information that may aid physicians in their decision process.

To address this need, we conducted the present study to evaluate effectiveness and tolerability in routine clinical practice of OW GLP-1RAs presently available in Italy, specifically dulaglutide (Eli Lily; FDA, 2014), OW exenatide (Astra Zeneca; FDA, 2005), and OW semaglutide (Novo Nordisk; FDA, 2017). Moreover, the metabolic effects of switching from a GLP-1RA to another were assessed.

## Methods

### Study Design, Data Source, and Participants

This was a retrospective real-world cohort study based on data routinely registered in an electronic chart system software (Smart Digital Clinic/METEDA, San Benedetto del Tronto, AP, Italy). It was created to assist Italian diabetes outpatient clinics in managing and reviewing patient data. The study adheres to the tenets of the Declaration of Helsinki and received the approval of the local ethics committee. Data of all patients with T2DM (at least a year since the onset of the disease) aged 18–80 years, who were first prescribed OW exenatide, dulaglutide, or OW semaglutide in the period between 2018 and 2021, were included. Patients with type 1, secondary, or gestational diabetes were excluded from the study.

Baseline visit (T0) was defined as the first prescription of dulaglutide, OW exenatide, or OW semaglutide with or without prior GLP-1RA therapy. At baseline, the following clinical and laboratory data were considered: age, sex, disease duration, height, body weight, body mass index (BMI), waist circumference (WC), HbA1c level, fasting plasma glucose (FPG), systolic blood pressures (SBP), diastolic blood pressures (DBP), triglycerides, total cholesterol, high-density lipoprotein cholesterol (HDL-C), low-density lipoprotein cholesterol (LDL-C), serum creatinine, estimated glomerular filtration rate (eGFR), and background diabetes therapy. Information on complications of diabetes, comorbidities, and concurrent use of lipid-lowering and antihypertensive medications was also collected.

Data availability is reported in **Appendix 1**.

Microangiopathy was defined as patients who had at least one of the following complications: neuropathy, retinopathy, macular edema, chronic kidney disease, or micro-albuminuria. Macroangiopathy was defined by the presence of any of the following: history of stroke/transient ischemic attack, history of myocardial infarction, ischemic heart disease, coronary artery disease, coronary revascularization, peripheral arterial disease, and peripheral revascularization.

Updated values of HbA1c and body weight were recorded also during follow-up visits (after 6 and 12 months, according to routine practice of the diabetes center). At each visit, adverse events, withdrawal reasons, and switching to another GLP-1RA were documented. There was no information on medicine prices or refill rates.

### Statistical Methods

Patients’ characteristics were reported as mean ± standard deviation (SD) or frequency and percentage and were compared between groups with Student’s t-test (or Mann–Whitney test, if appropriate) or Chi-square test for continuous and categorical variables, respectively.

To assess changes after 6 months (T6) and 12 months (T12) in continuous endpoints (HbA1c and body weight), longitudinal linear mixed models for repeated measures were applied. Results were expressed as estimated mean and estimated mean change from baseline with their 95% confidence intervals (CIs). Changes in HbA1c and body weight at T6 represented the co-primary endpoints.

Analyses were performed on the overall population and stratified by previous use of GLP1-RA (in patients with data on previous treatment available). Two subgroups were identified: GLP1-RA treatment naive patients (naive cohort) and patients who switched from another GLP1-RA (switchers cohort). No statistical comparisons were applied to these cohorts being independent and clinically different cohorts.

Furthermore, the effectiveness of the three long-acting GLP1-RA was compared overall and separately in the GLP1-RA naive and switchers cohorts. Multivariate analyses were run to take into account the effect of unbalanced covariates at baseline between patients treated with the different GLP1-RAs. In particular, a propensity score-based approach was used for confounding adjustment. Propensity scores were calculated with the Toolkit for Weighting and Analysis of Nonequivalent Groups (TWANG) package implemented in R software (version 3.5.2) and considering as potential confounders the unbalanced variables at baseline (age, gender, diabetes duration, HbA1c levels, body weight, eGFR, and use of concomitant glucose-lowering drugs). Propensity score was added as a covariate in the multivariate model in order to make a comparison between different GLP-1RAs within the cohort of switchers and naive. Statistical significance was defined for all the analyses as p < 0.05. Data were analyzed using SAS software (release 9.4; Cary, NC, USA).

## Results

### Participants’ Characteristics

A total of 1,001 patients with T2DM treated with an OW GLP-1RA prescribed between April 2018 and January 2021 were included in this study. Among these, 379 (37.9%) patients received semaglutide OW, 435 (43.4%) dulaglutide, or 187 exenatide OW (18.7%). Baseline patients’ characteristics overall and by type of long-acting GLP1-RA prescribed are reported in **Table 1**.

**Table 1:** Baseline patients characteristics overall and by GLP1-RA.

	Overall	Dulaglutide	Exenatide	Semaglutide	p-value*
N	1,001	435	187	379	
Age (years)	63.9 ± 9.2	64.9 ± 9.6	63.1 ± 8.9	63.1 ± 8.9	**0.006**
Gender (%)					
Women	43.9	43.0	53.5	40.1	**0.009**
Men	56.1	57.0	46.5	59.9	
Diabetes duration (years)	12.5 ± 7.9	13.4 ± 7.8	10.6 ± 7.2	12.4 ± 8.1	**0.0002**
Body weight (kg)	89.7 ± 18.3	87.4 ± 17.5	91.3 ± 18.4	91.7 ± 18.7	**0.004**
BMI (kg/m^2^)	32.8 ± 6.0	31.9 ± 5.5	34.2 ± 6.2	33.2 ± 6.4	**0.0001**
BMI ≥ 30 kg/m^2^	63.8	55.4	74.7	67.8	**<0.0001**
Waist circumference (cm)	110.4 ± 12.2	109.6 ± 12.0	111.5 ± 12.3	111.4 ± 12.7	0.20
Systolic blood pressure (mmHg)	140.3 ± 20.1	139.3 ± 18.6	152.0 ± 25.6	134.2 ± 16.0	**0.01**
Diastolic blood pressure (mmHg)	80.9 ± 10.3	79.4 ± 8.8	86.4 ± 14.7	80.2 ± 8.6	0.07
Fasting plasma glucose (mg/dl)	163.8 ± 41.6	164.6 ± 41.4	167.8 ± 35.9	159.1 ± 45.9	0.13
HbA1c (%)	8.1 ± 1.1	8.0 ± 1.0	7.8 ± 0.6	8.2 ± 1.3	**0.03**
Total cholesterol (mg/dl)	176.9 ± 41.1	176.8 ± 41.4	179.9 ± 35.7	175.6 ± 43.1	0.43
HDL Cholesterol (mg/dl)	45.1 ± 12.4	45.7 ± 12.8	47.5 ± 13.3	43.3 ± 11.3	**0.01**
Triglycerides (mg/dl)	180.7 ± 119.1	182.0 ± 129.8	169.1 ± 80.9	184.7 ± 122.2	0.87
LDL Cholesterol (mg/dl)	96.0 ± 34.3	96.2 ± 33.9	98.5 ± 29.7	94.8 ± 36.8	0.55
eGFR (mg/min/1.73 m^2^)	77.6 ± 22.7	74.0 ± 23.2	81.3 ± 18.1	79.4 ± 23.4	**0.01**
eGFR < 60 mg/min/1.73 m^2^ (%)	25.3	29.6	16.5	24.6	0.0575
Creatinine (mg)	1.0 ± 0.5	1.0 ± 0.4	0.9 ± 0.3	1.0 ± 0.6	0.07
Microalbuminuria (mg/dl)	95.7 ± 286.3	79.0 ± 239.4	98.6 ± 301.6	114.3 ± 327.6	0.55
Microangiopathy** (%)	39.3	36.5	43.1	39.6	0.37
Macroangiopathy*** (%)	22.8	25.0	16.5	24.3	0.07
Background glucose lowering treatments:					
Metformin (%)	82.2	83.2	86.1	79.2	0.10
Sulfonylureas (%)	12.4	16.3	18.7	4.7	**<0.0001**
Pioglitazone (%)	11.0	11.0	13.9	9.5	0.29
DPP4-inhibitors (%)	19.9	28.0	31.6	4.7	**<0.0001**
SGLT2-inhibitors (%)	10.7	12.9	8.6	9.2	0.14
Basal insulin therapy (%)	28.9	23.0	9.6	45.1	**<0.0001**
Antihypertensive treatment (%)	73.9	71.8	76.5	74.2	0.5147
Lipid-lowering treatment (%)	56.0	56.5	50.3	58.4	0.1829
Antiplatelet agents (%)	43.8	43.4	41.7	45.2	0.72
Switch from another GLP-1RA	36.6	25.9	16.0	58.0	**<0.0001**

Data are mean and standard deviations or proportion. *Student’s t-test, Mann–Whitney test, or Chi-square test as appropriate. Statistically significant p-values (p < 0.05) are in bold. **Retinopathy, nephropathy, and neuropathy ***Ischemic heart disease, stroke, and peripheral arterial disease.

Overall, 56.1% of patients were men, and mean (SD) age and diabetes duration were 63.9 (9.2) years and 12.5 (7.9) years, respectively. The mean (SD) HbA1c was 8.1% (1.1%) (or 65 mmol/mol), whereas average fasting glucose value was 164 mg/dl (or 9.11 mmol/L).

Most patients had one or more comorbidities, the most frequent of which were obesity (63.8%), hypertension (73.9%), and dyslipidemia (56.0%). Approximately 40% of patients had microangiopathy and <25% had macroangiopathy (**Table 1**).

Almost all patients had a background glucose-lowering therapy, specifically metformin (82.2%), basal insulin (28.9%), DPP-IV inhibitors (19.9%), sulfonylureas (12.4%), pioglitazone (11%), and SGLT-2 inhibitors (10.7%) (**Table 1**).

Furthermore, data on the previous treatment were available in 882 of 1,001 patients (88.1%). Among them, 323 (32.3%) were already treated with another GLP-1RA before starting one of the three OW GLP1-RAs of interest, whereas 559 (67.7%) were GLP1-RA naive. Baseline patients’ characteristics by naive or switchers cohort are reported in **Appendix 2**.

Patients starting the three OW GLP1-RAs significantly differed in terms of age, gender, diabetes duration, HbA1c levels, body weight, eGFR, and use of concomitant glucose-lowering drugs. These characteristics were taken into consideration in the propensity score adjustment.

Dulaglutide and OW exenatide were started with the recommended dose of 1.5 or 2 mg OW, respectively. OW semaglutide as add-on therapy was initiated at the recommended initial dose of 0.25 mg (52.5%). Patients were strictly followed in order to achieve an optimal metabolic control tailored on patients’ individual characteristics. Each GLP1-RAs was titrated to reach the maximum tolerated dose. In switchers, the starting dose was the higher available: 1.5 mg for dulaglutide and 2 mg for OW exenatide. In switchers who received semaglutide, the starting dose was 0.5 or 1.0 mg (36.4% and 11.1%, respectively) considering the higher dose tolerated by patients.

### Outcomes Data: Change in HbA1c and Body Weight From Baseline to 6 and 12 Months

Overall, after 6 months, mean change in HbA1c was −0.97% (95% C.I., −1.05;−0.89, *p* < 0.0001), and mean change in body weight was −2.64 kg (95% C.I., −3.05;−2.23, *p* < 0.0001). After 12 months, mean change in HbA1c was −0.89% (95% C.I., −0.98;−0.8, *p* < 0.0001), and the mean change in body weight was −2.63 kg (95% C.I., −3.08;−2.18, *p* < 0.0001) (**Table 2**).

**Table 2:** Changes in estimated mean levels of continuous endpoints during the follow-up by cohort and within-group group comparisons (T6 vs. T0 and T12 vs. T0).

Cohort	Endpoint	Visit	Estimated mean and 95% CI	Estimated mean difference from T0 and 95% CI	Within group p-value*
Overall	HbA1c	T0	8.05 (7.98;8.12)	–	–
		T6	7.08 (7.00;7.15)	−0.97 (−1.05; −0.89)	**<0.0001**
		T12	7.16 (7.08;7.25)	−0.89 (−0.98; −0.8)	**<0.0001**
	Weight	T0	89.67 (88.5;90.85)	–	–
		T6	87.03 (85.83;88.23)	−2.64 (−3.05; −2.23)	**<0.0001**
		T12	87.04 (85.82;88.25)	−2.63 (−3.08; −2.18)	**<0.0001**
Naive	HbA1c	T0	8.15 (8.06;8.24)	–	–
		T6	6.99 (6.9;7.09)	−1.16 (−1.26; −1.05)	**<0.0001**
		T12	7.09 (6.99;7.2)	−1.06 (−1.17; −0.95)	**<0.0001**
	Weight	T0	90.08 (88.53;91.64)	–	–
		T6	86.98 (85.41;88.55)	−3.11 (−3.54; −2.67)	**<0.0001**
		T12	86.8 (85.22;88.38)	−3.28 (−3.76; −2.81)	**<0.0001**
Switchers	HbA1c	T0	7.92 (7.82;8.02)	–	–
		T6	7.22 (7.1;7.34)	−0.70 (−0.82; −0.57)	**<0.0001**
		T12	7.29 (7.15;7.42)	−0.63 (−0.77; −0.49)	**<0.0001**
	Weight	T0	89.12 (87.31;90.93)	–	–
		T6	87.34 (85.44;89.25)	−1.78 (−2.64; −0.92)	**<0.0001**
		T12	87.72 (85.78;89.67)	−1.40 (−2.34; −0.45)	**0.004**

*Paired t-test derived from linear mixed models for repeated measurements. Statistically significant p-values (p < 0.05) are in bold.

In the naive cohort, HbA1c levels were reduced of over 1% and body weight of over 3 kg at T6, whereas in the switchers cohort, HbA1c levels were reduced by −0.7% and body weight by −1.7 kg. Benefits were substantially maintained in both cohorts at T12.

The propensity-score-adjusted comparisons in the effectiveness of the three OW GLP1-RAs are reported in **Appendix 3** (overall cohort) and **Table 3** (naive and switchers cohorts).

**Table 3:** Changes in propensity score adjusted estimated mean levels of continuous endpoints during the follow-up by cohort and GLP-1RA.

Endpoint	GLP-1RA	Visit	NAIVE				SWITCHERS			
			Estimated mean and 95% CI	Estimated mean difference from T0 and 95% CI	Within group p-value*	Between group p-value**	Estimated mean and 95% CI	Estimated mean difference from T0 and 95% CI	Within group p-value*	Between group p-value**
**HbA1c**	**Dulaglutide**	T0	8.11 (7.98;8.24)	–	–		7.89 (7.74;8.05)			
		T6	7.08 (6.94;7.22)	−1.03 (−1.18;−0.88)	**<0.0001**	**0.001**	7.29 (7.12;7.47)	−0.60 (−0.79;−0.41)	**<0.0001**	0.09
		T12	7.18 (7.03;7.33)	−0.93 (−1.09;−0.77)	**<0.0001**	**0.0006**	7.4 (7.22;7.59)	−0.49 (−0.69;−0.29)	**<0.0001**	0.06
	**Exenatide**	T0	7.87 (7.71;8.04)	–	–		7.46 (7.08;7.83)			
		T6	6.88 (6.71;7.05)	−1.00 (−1.18;−0.81)	**<0.0001**		7.04 (6.64;7.44)	−0.42 (−0.85;0.01)	0.05	
		T12	6.94 (6.75;7.12)	−0.94 (−1.14;−0.74)	**<0.0001**		7.05 (6.64;7.46)	−0.41 (−0.84;0.03)	0.07	
	**Semaglutide**	T0	8.51 (8.34;8.68)	–	–		8.00 (7.87;8.14)			
		T6	6.95 (6.75;7.16)	−1.55 (−1.77;−1.34)	**<0.0001**		7.16 (6.98;7.35)	−0.84 (−1.03;−0.65)	**<0.0001**	
		T12	6.96 (6.7;7.22)	−1.55 (−1.82;−1.28)	**<0.0001**		7.14 (6.91;7.37)	−0.87 (−1.1;−0.63)	**<0.0001**	
**Weight**	**Dulaglutide**	T0	89.21 (86.93;91.49)	–	–		85.57 (82.88;88.27)			
		T6	86.5 (84.2;88.79)	−2.71 (−3.42;−2.01)	**<0.0001**	0.15	84.39 (81.64;87.14)	−1.18 (−2.13;−0.24)	**0.01**	**0.0006**
		T12	86.77 (84.46;89.08)	−2.44 (−3.2;−1.68)	**<0.0001**	**0.0006**	84.93 (82.17;87.69)	−0.64 (−1.61;0.33)	0.19	0.14
	**Exenatide**	T0	90.39 (87.48;93.29)	–	–	–	96.24 (89.76;102.72)			
		T6	87.09 (84.18;90.01)	−3.29 (−4.09;−2.49)	**<0.0001**		96.03 (89.52;102.54)	−0.21 (−2.03;1.62)	0.82	
		T12	86.74 (83.81;89.68)	−3.64 (−4.52;−2.77)	**<0.0001**		95.78 (89.24;102.31)	−0.46 (−2.37;1.46)	0.64	
	**Semaglutide**	T0	91.07 (88.04;94.09)	–	–		90.79 (88.24;93.34)			
		T6	87.31 (84.11;90.5)	−3.76 (−5.05;−2.47)	**<0.0001**		87.36 (84.61;90.11)	−3.43 (−4.67;−2.19)	**<0.0001**	
		T12	84.78 (81.43;88.13)	−6.29 (−7.94;−4.63)	**<0.0001**		87.24 (84.28;90.19)	−3.55 (−5.2;−1.91)	**<0.0001**	

Within-group and between-group comparisons (T6 vs. T0 and T12 vs. T0). *Paired t-test derived from linear mixed models for repeated measurements. **Unpaired t-test derived from linear mixed models for repeated measurements. Statistically significant p-values (p<0.05) are in bold. *Chi-square test between, OW exenatide, and OW semaglutide. Statistically significant p-values (p < 0.05) are in bold.

For both endpoints (HbA1c and body weight), in the overall population, statistically significant improvements were obtained with all the three OW GLP1-RAs at T6 and at T12, with semaglutide showing the largest effect (−1.14% (95% C.I., −1.29;−0.99, *p* < 0.0001) in HbA1c and −3.55 kg (95% C.I., −4.45;−2.65; *p* < 0.0001) in body weight at T6, substantially maintained at T12 (**Appendix 3**).

Even stratifying the overall population by switchers and naive, semaglutide was confirmed to produce the largest reduction in HbA1c and weight from baseline both at T6 and T12 in both cohorts, followed by dulaglutide. The effectiveness of exenatide was documented in the naive cohort but not in the switchers’ cohort (**Table 3**).

**Figure 1:**
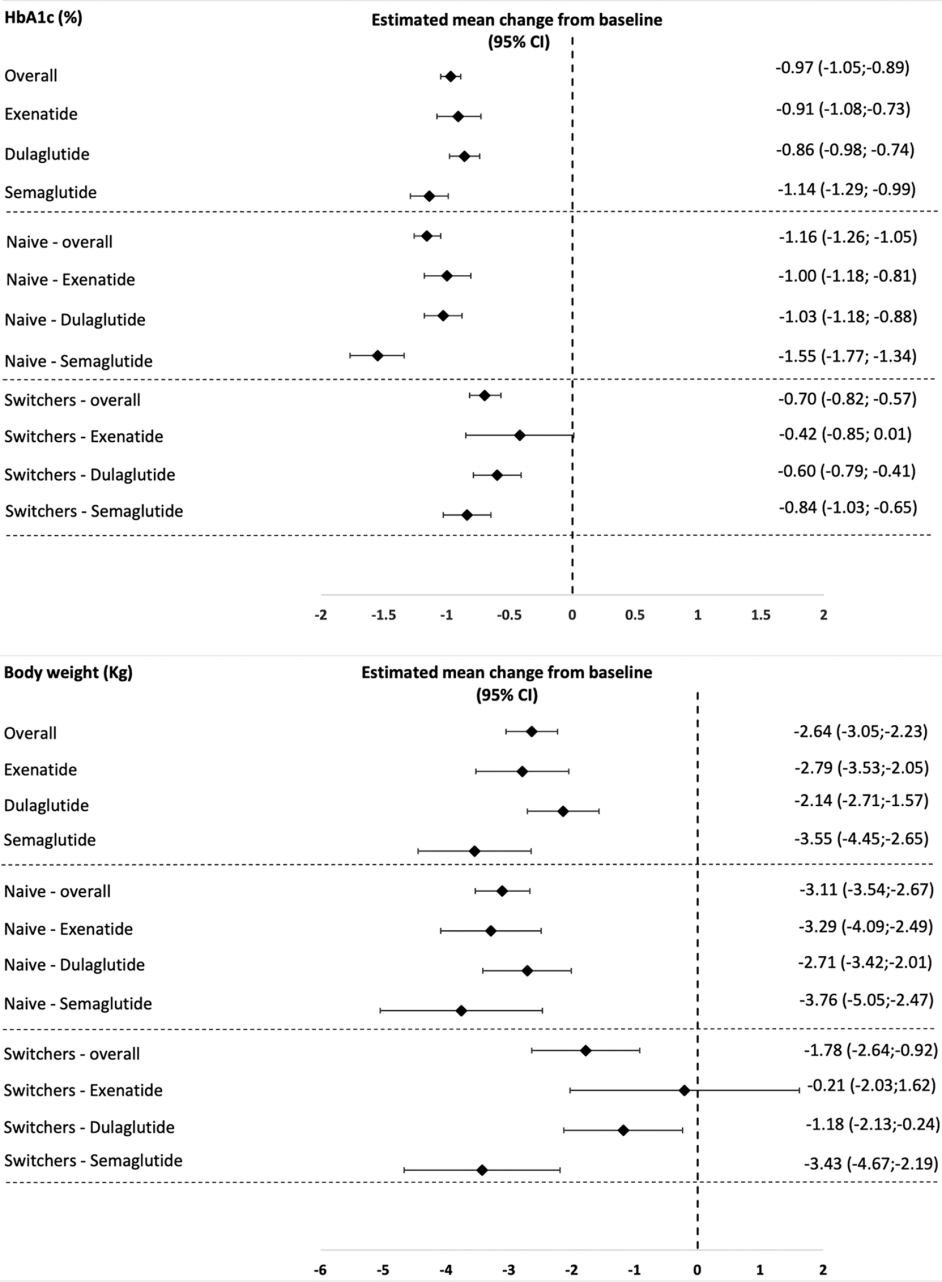
Estimated mean changes from baseline in HbA1c and body weight in the different study populations.

Estimated mean changes from baseline and their 95% confidence intervals of HbA1c and body weight in the different study populations are reported in **Figure 1**.

No changes in concomitant glucose-lowering drugs occurred during 12 months.

### Safety Endpoints: Adverse Events and Discontinuation Rate

The overall proportion of patients reporting adverse events was 10.0%, with a significant difference between dulaglutide (10.3%), OW exenatide (16%), and OW semaglutide (6.6%). Gastrointestinal side effects were the most frequent adverse events (7.8%) and occurred in higher proportions of patients receiving dulaglutide (10.1%) and exenatide (7.5%) than semaglutide (5.8%). Injection site reactions such as subcutaneous nodules were reported mainly in patients receiving exenatide OW (7.5%) and in a small minority of patients treated with dulaglutide (0.2%) and semaglutide (0.8%). No cases of severe hypoglycemia, pancreatitis, or thyroid cancer were reported (**Table 4**).

**Table 4:** Safety endpoints overall and by cohort.

	Overall	Naive	Switchers	Dulaglutide	Exenatide	Semaglutide	p-value
N	1001	559	323	435	187	379	
* Adverse events*	100 (10.0)	77 (13.8)	16 (5.0)	45 (10.3)	30 (16.0)	25 (6.6)	**0.002**
Type of adverse events							
* Headache*	2 (0.002)	2 (0.4)	0 (0.0)	0 (0.0)	2 (1.0)	0 (0.0)	**<0.0001**
* Gastrointestinal side effects*	80 (7.8)	61 (10.9)	12 (3.7)	44 (10.1)	14 (7.5)	22 (5.8)	
* Injection site reactions*	18 (1.8)	14 (2.5)	4 (0.3)	1 (0.2)	14 (7.5)	3 (0.8)	
Drop-out	237 (23.7)	160 (28.6)	52 (16.1)	124 (28.5)	78 (41.7)	35 (9.2)	**<0.0001**
Causes of dropout							
* Headache*	2 (0.8)	2 (1.3)	0 (0.0)	0 (0.0)	2 (2.6)	0 (0.0)	**<0.0001**
* Gastrointestinal side effects*	66 (27.8)	52 (32.5)	9 (17.3)	37 (29.8)	14 (17.9)	15 (42.9)	
* Injection site reactions*	17 (7.2)	13 (8.1)	4 (7.7)	1 (0.8)	14 (17.9)	2 (5.7)	
* Patient decision*	42 (18.3)	27 (17.1)	10 (20.8)	24 (20.3)	11 (14.1)	7 (21.2)	
* Need of therapy intensification*	78 (34.1)	54 (34.2)	18 (37.5)	37 (31.4)	34 (43.6)	7 (21.2)	
* Hospital admission for all causes*	24 (10.5)	10 (6.3)	7 (14.6)	19 (16.1)	3 (3.8)	2 (6.1)	
* Unknown*	8 (3.4)	2 (1.3)	4 (7.7)	6 (4.8)	0 (0.0)	2 (5.7)	

Data are frequency and proportion. *Chi-square test between dulaglutide, OW exenatide, and OW semaglutide. Statistically significant p-values (p < 0.05) are in bold.

During the entire follow-up period, 237 (23.7%) patients discontinued treatment with a median time (interquartile range) to treatment discontinuation of 279.3 (214.3) days. The lowest proportion of dropout was found in the semaglutide group (**Table 4**).

Overall, the main reasons for discontinuation were limited effectiveness in lowering HbA1c and/or body weight and need of therapy intensification (34.1%), gastrointestinal side effects (28.8%), patient’s decision (18.3%), and not treatment-related hospitalizations (10.5%).

As expected, dropout patients were more frequently naive than switchers, reported more frequently AEs, and were often treated with additional oral agents other than metformin (data not shown).

## Discussion

OW GLP-1RAs (i.e., dulaglutide, OW exenatide, and OW semaglutide) were proven to give benefits on glucose control, body weight reduction, cardiovascular risk factors, and outcomes in subjects with T2D as shown by previous RCTs and CVOT data (16). Real-world studies are playing an increasingly important role in clinical practice and physicians’ decision making. In this context, real-world data on switching between GLP-1RAs are limited.

In this single-center, retrospective study, 1,001 patients treated with the available OW GLP-1RAs (dulaglutide, OW exenatide, and OW semaglutide) experienced statistically significant and clinically relevant reductions in both HbA1c and body weight.

As to the results obtained with each molecule, dulaglutide determined a mean reduction in HbA1c of −0.85% and −0.74% and body weight of −2.13 and −1.75 kg (observations at 6 and 12 months, respectively). These results are in line with the findings of the large AWARD program, where both doses of 0.75 and 1.5 mg once-weekly induced HbA1c reduction between −0.7% and –1.6% as monotherapy or in combination (dual or triple therapy). In AWARD studies, dulaglutide use was associated with a sustained weight loss between −1.5 and –3 kg (10, 17–24). In a real-world setting, including 1,307 subjects, dulaglutide reduced HbA1c by −1.0% and body weight by −2.9 kg after 30 months of follow-up, also in this case in line with our observations (25).

In patients started on OW exenatide, HbA1c and body weight were reduced from baseline by −0.92% and −0.79% and by −2.78 and −2.94 kg at 6 and 12 months respectively. These data are in line with previous observations (25–32). The DURATION trials, testing OW exenatide (in several clinical situations including add-on to pioglitazone, sitagliptin, metformin, and insulin glargine), documented reductions in HbA1c of −1.3 to −1.9% points and body weight reductions in the range of −2.0 to −3.7 kg from baseline associated with the use of this molecule (33–39).

The largest reductions in HbA1c and body weight (−1.14% and −1.15%;−3.55 and −4.92 kg after 6 and 12 months, respectively) were observed in our study in patients who were started on OW semaglutide. These are consistent with those observed in the SUSTAIN program, where the change in HbA1c ranged from − 1.1% to − 1.8% points and change in body weight ranged from −3.5 to −6.5 kg with OW semaglutide versus comparators (placebo, sitagliptin, exenatide extended-release, insulin glargine, dulaglutide, canagliflozin, and liraglutide) (9, 11, 40–47). Few studies investigated OW semaglutide in a real-world setting (48, 49). Recently, the SURE program evaluated the use of OW semaglutide in populations of T2DM patients from four different countries (50–53): our findings are comparable with those obtained in the SURE program, even if patients enrolled in the SURE program had higher basal HbA1c and BMI as compared to our population.

A similar paper recently published by Di Loreto and collaborators confirmed effectiveness and tolerability of semaglutide in clinical practice, irrespective of diabetes duration and severity (54). It should be emphasized that, compared to this work, our analysis is based on a wider case history and has the advantage of analyzing the weekly GLP1-RAs currently on the market in Italy.

GLP-1RAs treatment might be discontinued due to gastrointestinal adverse events or lack of efficacy. Switching between GLP-1RAs represents a possible choice when the ongoing drug is unable to provide the desired glycemic control and/or weight loss or when gastrointestinal adverse effects occur (7, 8). Indeed, exposure–response modeling suggests that switching to OW semaglutide from OW exenatide, dulaglutide, or liraglutide allows to achieve further reductions in HbA1c and body weight (7). This would be consistent with the few available real-world observations on switching to semaglutide from other GLP-1RAs (8, 55–57).

In our study, one-third of the patients were previously treated with another GLP-1RAs (switchers). Thus, we compared the effectiveness of dulaglutide, OW exenatide, and OW semaglutide in switchers and patients naive to GLP-1RAs through a propensity score adjustment.

We found that HbA1c and body weight in the switchers’ cohort further improved over time. In particular, OW semaglutide was effective in ameliorating HbA1c and body weight in both naive and switcher patients in a more durable manner (12 months). The effectiveness of dulaglutide and OW exenatide was prominent in the naive cohort and still present in the switchers cohort, although to a lesser extent as compared to OW semaglutide. Hence, our study supports the practice of switching treatment between different GLP-1RA in order to achieve further reductions in HbA1c and body weight. The benefit of doing so seems to be particularly evident when switching to OW semaglutide.

Thus, our results support what was suggested in the Expert Consensus authored by Akshay and colleagues, where HbA1c is not on target; the need for additional weight loss and cardiovascular and renal benefits are described as triggers for switching (58).

We observed a good persistence in therapy with all three OW GLP-1RAs investigated, even though a significant heterogeneity between molecules emerged. OW semaglutide was associated with the lowest discontinuation rate (9.2%), and dulaglutide discontinuation rate (28.5%) was definitely lower as compared to OW exenatide (41.7%). Dulaglutide appeared to be slightly more prescribed in older patients with a longer diabetes duration probably for the easier management of the device, which could improve the compliance to therapy. Our results are at odds with data published by Mody and collaborators who, in a larger population, found better performance in terms of adherence and persistence to therapy with dulaglutide as compared to OW semaglutide and OW exenatide in a larger population (12). The difference in data sources (administrative vs. clinical) might contribute to explaining the discrepancy in the results.

This study has strengths and limitations. To the best of our knowledge, this is the first Italian retrospective real-world study considering all the available OW GLP-1RAs in a routine clinical practice with OW semaglutide included in the analysis. Among the limitations, given the restrictions associated with the coronavirus disease 2019 (COVID-19) outbreak, telemedicine was implemented to provide diabetes care and replaced, unavoidably, in-person visits. Therefore, some follow-up data regarding body weight were missing or self-reported. However, the analysis of HbA1c change over time was not affected because biochemical monitoring continued over this period.

In conclusion, based on our results, the effectiveness and tolerability of available OW GLP-1RAs (dulaglutide, OW exenatide, and OW semaglutide) in a routine clinical practice setting are confirmed. Moreover, our data indicate that when patients are not responding to a given GLP-1RA, switching to another OW GLP-1RA is a viable option that might help in achieving metabolic targets and in gaining additional clinical benefits. Among OW GLP-1RAs, semaglutide seems to be more effective on HbA1c and weight reduction as compared to OW exenatide dulaglutide used at the maximal doses presently available in Italy.

## Data Availability Statement

The raw data supporting the conclusions of this article will be made available by the authors, without undue reservation.

## Ethics Statement

The studies involving human participants were reviewed and approved by Comitato Etico per la Ricerca Biomedica delle Province di Chieti e Pescara e dell’Università degli Studi “G. d’Annunzio” di Chieti e Pescara. Written informed consent for participation was not required for this study in accordance with the national legislation and the institutional requirements.

## Author Contributions

GD, SC, AG, BC, MM, and FC were responsible for data collection. MR, GG, and AN performed data analysis and contributed to data interpretation and tables/figures drafting. GD, SC, MB, and AG are responsible for data interpretation and manuscript drafting. GF and AC were responsible for study design, results interpretation, and manuscript drafting and revision. All authors contributed to the article and approved the submitted version.

## Funding

No funding or sponsorship was received by the authors for this study. Novo Nordisk S.p.A. provided an unconditional grant to CORESEARCH SRL (MR, GG, and AN) for the support in the analysis. The authors of the publication are fully responsible for the contents and conclusions. Novo Nordisk S.p.A. was not involved in the study design, collection, analysis, interpretation of data, the writing of this article or the decision to submit it for publication.

## Conflict of Interest

GG, MR, and AN are employed by CORESEARCH SRL.

The remaining authors declare that the research was conducted in the absence of any commercial or financial relationships that could be construed as a potential conflict of interest.

## Publisher’s Note

All claims expressed in this article are solely those of the authors and do not necessarily represent those of their affiliated organizations, or those of the publisher, the editors and the reviewers. Any product that may be evaluated in this article, or claim that may be made by its manufacturer, is not guaranteed or endorsed by the publisher.
