# Supplementary material for: Effectiveness and Tolerability of Once-Weekly GLP-1 Receptor Agonists in Clinical Practice: A Focus on Switching Between Once-Weekly Molecules in Type 2 Diabetes
Source: Front Endocrinol (Lausanne). 2022 Jul 15;13:892702. doi: 10.3389/fendo.2022.892702 (PMC9335857; doi:10.3389/fendo.2022.892702)
Supplement: Supplementary file 1 [file DataSheet_1.docx]

Supplementary Material

**Appendix 1: Data availability**

| Variable | **Overall** | **Naive** | **Switchers** | **Dulaglutide** | **Exenatide** | **Semaglutide** |
| --- | --- | --- | --- | --- | --- | --- |
| **N** | 1001 | 559 | 323 | 435 | 187 | 379 |
| Age | 1001 | 559 | 323 | 435 | 187 | 379 |
| Gender | 1001 | 559 | 323 | 435 | 187 | 379 |
| Diabetes duration | 981 | 546 | 320 | 424 | 185 | 372 |
| Body weight at T0 | 903 | 516 | 279 | 392 | 182 | 329 |
| Body weight at T6 | 550 | 369 | 147 | 279 | 165 | 106 |
| Body weight at T12 | 432 | 289 | 113 | 238 | 135 | 59 |
| BMI | 903 | 516 | 279 | 392 | 182 | 329 |
| Waist circumference | 591 | 384 | 149 | 318 | 161 | 112 |
| Systolic blood pressure | 111 | 64 | 29 | 62 | 20 | 29 |
| Diastolic blood pressure | 111 | 64 | 29 | 62 | 20 | 29 |
| Fasting plasma glucose | 365 | 222 | 94 | 186 | 79 | 100 |
| HbA1c at T0 | 991 | 558 | 322 | 427 | 187 | 377 |
| HbA1c at T6 | 718 | 442 | 216 | 336 | 173 | 209 |
| HbA1c at T12 | 558 | 350 | 159 | 293 | 143 | 122 |
| Total Cholesterol | 575 | 308 | 193 | 245 | 105 | 225 |
| HDL Cholesterol | 551 | 295 | 191 | 235 | 101 | 215 |
| Triglycerides | 570 | 311 | 189 | 237 | 105 | 228 |
| LDL Cholesterol | 531 | 284 | 185 | 222 | 97 | 212 |
| eGFR | 557 | 286 | 197 | 216 | 85 | 256 |
| Creatinine | 557 | 286 | 197 | 216 | 85 | 256 |
| Microalbuminuria | 254 | 172 | 79 | 106 | 63 | 85 |
| Microangiopathy* | 776 | 399 | 266 | 282 | 181 | 313 |
| Macroangiopathy** | 812 | 423 | 276 | 272 | 182 | 358 |
| Glucose-lowering drugs | 1001 | 559 | 323 | 435 | 187 | 379 |
| Antihypertensive treatment | 850 | 443 | 290 | 287 | 187 | 376 |
| Lipid-lowering treatment | 847 | 442 | 289 | 285 | 187 | 375 |
| Antiplatelet agents | 847 | 442 | 289 | 286 | 187 | 374 |
| Dose GLP1-RA | 1001 | 559 | 323 | 435 | 187 | 379 |

*Data express N of valid values for each variable. *Retinopathy, nephropathy, and neuropathy **Ischemic heart disease, stroke, and peripheral arterial disease.*

**Appendix 2: Baseline patients’ characteristics by cohort**

|  | **Naive** | **Switchers** |
| --- | --- | --- |
| **N** | 559 | 323 |
| Age (years) | 63.1±9.4 | 64.2±8.6 |
| Gender (%) |  |  |
| Women | 43.1 | 46.4 |
| Men | 56.9 | 53.6 |
| Diabetes duration (years) | 11.4±7.7 | 14.1±7.8 |
| Body weight (Kg) | 89.9±18.3 | 90.4±18.3 |
| BMI (kg/m^2^) | 32.8±5.9 | 33.3±6.4 |
| Waist circumference (cm) | 110.1±12.2 | 112.0±12.3 |
| Systolic blood pressure (mmHg) | 143.9±22.1 | 135.3±18.8 |
| Diastolic blood pressure (mmHg) | 82.4±12.2 | 79.0±7.3 |
| Fasting plasma glucose (mg/dl) | 172.3±43.9 | 150.4±31.2 |
| HbA1c (%) | 8.2±1.1 | 7.8±1.0 |
| Total Cholesterol (mg/dl) | 181.1±41.3 | 172.0±37.9 |
| HDL Cholesterol (mg/dl) | 45.3±12.6 | 44.9±11.8 |
| Triglycerides (mg/dl) | 178.1±120.1 | 184.0±119.4 |
| LDL Cholesterol (mg/dl) | 101.4±35.8 | 90.8±31.2 |
| eGFR (mg/min/1.73 m^2^) | 79.3±22.3 | 77.6±22.3 |
| eGFR <60 mg/min/1.73 m^2^ (%) | 21.7 | 26.9 |
| Creatinine (mg) | 1.0±0.6 | 1.0±0.4 |
| Microalbuminuria (mg/dl) | 90.9±264.7 | 103.9±332.2 |
| Microangiopathy* (%) | 39.1 | 41.7 |
| Macroangiopathy** (%) | 22.7 | 23.9 |
| Background glucose lowering treatments: |  |  |
| Metformin (%) | 82.1 | 85.4 |
| Sulfonylureas (%) | 14.1 | 9.9 |
| Pioglitazone (%) | 12.0 | 10.2 |
| DPP4-inhibitors (%) | 30.6 | 0.6 |
| SGLT2-inhibitors (%) | 15.7 | 0.6 |
| Basal insulin therapy (%) | 24.5 | 35.3 |
| Antihypertensive treatment (%) | 70.9 | 76.2 |
| Lipid-lowering treatment (%) | 52.3 | 58.1 |
| Antiplatelet agents (%) | 41.6 | 46.7 |

*Data are mean and standard deviations or proportion. *Retinopathy, nephropathy, and neuropathy **Ischemic heart disease, stroke, and peripheral arterial disease.*

**Appendix 3: Changes in propensity score adjusted estimated mean levels of continuous endpoints during the follow-up by GLP-1RA. Within-group and between-group group comparisons (T6 vs. T0 and T12 vs. T0).**

|  |  |  | **OVERALL** | | | |
| --- | --- | --- | --- | --- | --- | --- |
| **Endpoint** | **GLP-1RA** | **Visit** | **Estimated mean and 95% CI** | **Estimated mean difference from T0 and 95% CI** | **Within group p-value*** | **Between group**  **p-value**** |
| **HbA1c** | **Dulaglutide** | T0 | 8.02  (7.92;8.12) | - | - | - |
|  |  | T6 | 7.16  (7.05-7.27) | -0.86  (-0.98; -0.74) | **<0.0001** | **<0.0001** |
|  |  | T12 | 7.27  (7.15;7.39) | -0.75  (-0.88; -0.63) | **<0.0001** | **<0.0001** |
|  | **Exenatide** | T0 | 7.81  (7.65;7.96) | - | - |  |
|  |  | T6 | 6.9  (6.74;7.06) | -0.91  (-1.08; -0.73) | **<0.0001** |  |
|  |  | T12 | 6.95  (6.78;7.13) | -0.85  (-1.03; -0.67) | **<0.0001** |  |
|  | **Semaglutide** | T0 | 8.20  (8.09;8.31) | - | - |  |
|  |  | T6 | 7.06  (6.92;7.2) | -1.14  (-1.29; -0.99) | **<0.0001** |  |
|  |  | T12 | 7.05  (6.87;7.22) | -1.15  (-1.34; -0.97) | **<0.0001** |  |
| **Weight** | **Dulaglutide** | T0 | 87.7  (85.95;89.44) |  | - | - |
|  |  | T6 | 85.56  (83.79;87.33) | -2.14  (-2.71; -1.57) | **<0.0001** | **<0.0001** |
|  |  | T12 | 85.96  (84.17;87.74) | -1.74  (-2.35; -1.13) | **<0.0001** | **<0.0001** |
|  | **Exenatide** | T0 | 91.29  (88.61;93.96) |  | - |  |
|  |  | T6 | 88.49  (85.81;91.18) | -2.79  (-3.53; -2.05) | **<0.0001** |  |
|  |  | T12 | 88.19  (85.49;90.89) | -3.1  (-3.9; -2.3) | **<0.0001** |  |
|  | **Semaglutide** | T0 | 90.95  (88.98;92.92) |  | - |  |
|  |  | T6 | 87.4  (85.3;89.5) | -3.55  (-4.45; -2.65) | **<0.0001** |  |
|  |  | T12 | 86.03  (83.79;88.26) | -4.92  (-6.1; -3.74) | **<0.0001** |  |

**Paired t-test derived from linear mixed models for repeated measurements. **Unpaired t-test derived from linear mixed models for repeated measurements. Statistically significant p-values (p<0.05) are in bold.*
